# Low HDL cholesterol is correlated to the acute ischemic stroke with diabetes mellitus

**DOI:** 10.1186/1476-511X-13-171

**Published:** 2014-11-14

**Authors:** Yun Luo, Jingwei Li, Junfeng Zhang, Yun Xu

**Affiliations:** Department of Neurology, Affiliated Drum Tower Hospital of Nanjing University Medical School, Nanjing, 210008 China; School of Life Science, Nanjing University, Nanjing, 210093 China

**Keywords:** Diabetes mellitus, Ischemic stroke, High density lipoprotein cholesterol

## Abstract

**Background:**

To clarify the role of lipid composition in the occurrence of acute ischemic stroke (AIS) with diabetes mellitus (DM) and its influence factors.

**Methods:**

Data was collected from the patients hospitalization in Affiliated Drum Tower Hospital of Nanjing University Medical School from October 2008 to May 2012, which included AIS and non-AIS consist of transient ischemic attack (TIA) and Vertigo or dizzy. Lipid and other risk factors including blood glucose (BG), uric acid (UA), hypertension, DM and atrial fibrillation (AF) were investigated in relation to occurrence of AIS.

**Results:**

The level of high density lipoprotein (HDL) cholesterol was decreased obviously in the DM group compared to the non-DM group and low level of HDL cholesterol was prevalent in the AIS patients with DM. logistic regression demonstrated that decreased HDL cholesterol was correlated to the AIS with DM, not all AIS, and the relative risk of ischemic stroke in low HDL cholesterol level group was 2.113 (95% CI = 1.191-3.749, P = 0.011) compared to the high level group. Furthermore, age has the obviously impact on it. HDL cholesterol was correlated to the AIS with DM just in the populations of aged ≦70 years (OR = 0.192, P = 0.000), low level of HDL cholesterol had more high risk of ischemic stroke than that in the high level group (OR = 6.818, P = 0.002).

**Conclusion:**

Decreased HDL cholesterol was correlated to the occurrence of AIS with DM, especially in the populations of aged ≦70 years.

## Introduction

Diabetes mellitus (DM) is considered as one of the important risk factors of acute ischemic stroke (AIS), which has been proved in a series of studies including our previous report [1]. The important pathogenesy behind it is atherosclerosis (AS) [2, 3], which perhaps has more direct correlation with the blood lipid [4]. As the independent risk factors for cardiovascular and cerebrovascular disease, both of the DM and blood lipid have the impact on the AIS which is accepted wildly, but the relation between the DM and lipid during the occurrence and development of AIS is unclear.

In the previous studies about AS and AIS, among the lipid compositions, more attentions were paid to the role of low density lipoprotein cholesterol (LDL cholesterol). High level of LDL cholesterol was considered to be a predictor of cardiovascular and cerebrovascular diseases(CVD) in the general population. But in the past several years recently, the role of high density lipoprotein cholesterol (HDL cholesterol) was continued to be known. The relation between HDL cholesterol and the risk of ischemic stroke was inconsistent. In the current opinions, more studies supported an inverse association between HDL cholesterol and the risk of ischemic stroke [5, 6], despite this was not confirmed in the populations of Asia [7].

Diabetic individuals, as a special population with a 2- to 3-fold increase in the risk of stroke than the general population, controlling of the blood glucose failed to reduce the incidence of AIS [8–10]. If there any things can we do to prevent the occurrence of AIS, risk factors for AIS in diabetic individuals were not fully known until now. Patients with DM have significantly increased serum triglyceride (TG) and decreased HDL cholesterol concentrations, especially the latter [11], which plays a major role in the progression of atherosclerosis [6] (This also has been confirmed in our previous article, in Chinese). Based on this background, we hypothesis that HDL cholesterol may participate in the course of AIS induced by DM. The purpose of present study was to evaluate the role of HDL cholesterol on the risk of AIS events in the population of DM and its relative influence factors.

## Materials and methods

### Study subjects

Data for this retrospective study was collected from the hospitalization patients of department of neurology in Affiliated Drum Tower Hospital of Nanjing University Medical School from October 2008 to May 2012, which included AIS within 7 days of symptom onset, transient ischemic attack (TIA) and vertigo or dizzy. Here, both of the latter were set as the control represented the non-AIS population. The study was approved by institutional committee of Affiliated Drum Tower Hospital of Nanjing University Medical School. Patients who were found with pre-stroke impairment or insulin-dependent diabetes mellitus and vertigo or dizzy with organic diseases were excluded. At admission, plain CT scan of the head was done to rule out haemorrhage and MRI was done to identify the new infarction, otherwise such patients would also be excluded.

### Definition of vascular risk factors

Hypertension and diabetes mellitus were defined as participants with history of relative disease or new diagnosis according to the China hypertension and DM standard (just non-insulin-dependent diabetes were included), while atrial fibrillation (AF) was defined as participants with history of AF or new diagnosis by electrocardiogram.

### Blood collection and analysis

Venous blood was collected following overnight fasting for at least 12 hours, and analyzed by a solid-phase chemiluminescent immunometric assay on Immulite 2000 with the manufacturer’s reagents as directed to detect blood glucose (BG), uric acid (UA), Triglyceride (TG), total cholesterol (TC), High density lipoprotein cholesterol (HDL cholesterol), Low density lipoprotein cholesterol (LDL cholesterol).

### Statistical analyses

Statistical analyses were performed with SPSS 10.0 software. The results are expressed as constituent ratio for categorical variables (χ2 test) and as mean ± SEM for the continuous variables (t-test) depending on their normal distribution. The level-risk relationship was expressed as an OR, with a corresponding 95% CI, through logistic regression. Level of significance for statistical purposes was stated at p < 0.05.

## Results

### Baseline characteristics

610 patients with AIS were included in the trial, among them, 385 were male and 225 were female, whose age range from 15 to 92. 202 patients had DM, 434 patients had hypertension and 81 patients had AF coexistence with AIS. While 225 non-AIS patients were set as the control group, which contained 59 TIA patients and 166 patients with vertigo or dizziness, with age- and sex-comparable to the AIS group.

### Low level of HDL cholesterol prevalence in the DM group

**Figure 1:**
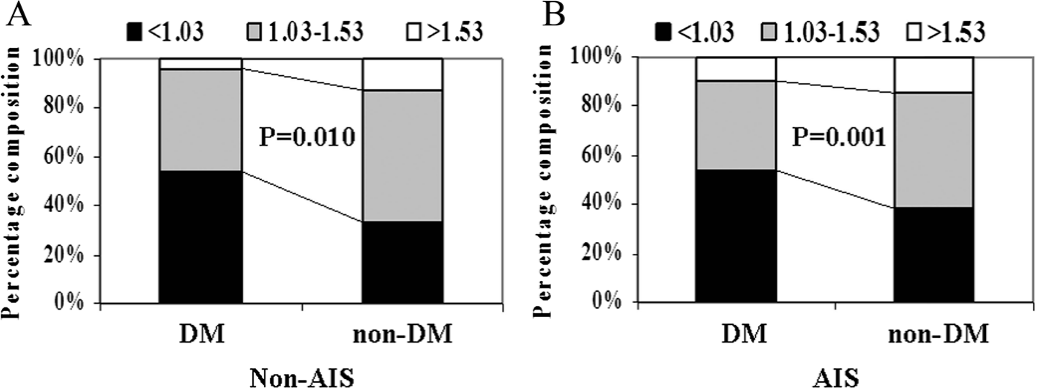
**Distribution of HDL cholesterol levels in the analyzed population. (A)** In the populations of Non-AIS. **(B)** In the populations of AIS.

HDL cholesterol concentrations were grouped into 3 levels: <1.03, 1.03-1.53, >1.53 mmol/l. We analyzed the difference of distribution of HDL cholesterol between the DM and non-DM group whenever in the AIS or non-AIS patients (we did not divide it into TIA and vertigo or dizzy for too small samples), and found that, the percent of low HDL cholesterol level was higher in DM group than that in non-DM group. The exact value was 54% vs 34% in the non-AIS patients and 53% vs 38% in the AIS patients, and both of the p value was below 0.05 (Figure 1).

### Lipid levels in AIS patients and controls

The level of HDL cholesterol was decreased obviously in the DM group compared to the non-DM group in the whole population, so did it in the AIS patients or AIS without AF patients. But in the non-AIS patients, there only has the trend of decline (P = 0.061), which mainly came from the populations of TIA (P = 0.038) (Table 1).

**Table 1:** **Comparison of lipid composition between with or without DM of the AIS or non**-**AIS patients**

Patients	Group	TG(mmol/l)	TC(mmol/l)	HDL-C(mmol/l)	LDL-C(mmol/l)
	non-DM(n = 408)	1.470 ± 0.050	4.812 ± 0.048	1.182 ± 0.019	2.549 ± 0.035
AIS(n = 610)	DM(n = 202)	1.582 ± 0.066	4.774 ± 0.080	1.067 ± 0.023	2.591 ± 0.058
	P value	0.190	0.673	0.000	0.538
AIS without	non-DM(n = 350)	1.532 ± 0.056	4.803 ± 0.052	1.159 ± 0.019	2.551 ± 0.038
AF(n = 529)	DM(n = 179)	1.647 ± 0.072	4.832 ± 0.084	1.056 ± 0.023	2.638 ± 0.061
	P value	0.220	0.757	0.001	0.206
Vertigo or	non-DM(n = 131)	1.442 ± 0.073	4.702 ± 0.089	1.163 ± 0.028	2.460 ± 0.070
Dizziness	DM(n = 35)	1.487 ± 0.098	4.359 ± 0.127	1.109 ± 0.059	2.237 ± 0.091
(n = 166)	P value	0.765	0.064	0.391	0.122
	non-DM(n = 38)	1.519 ± 0.169	4.251 ± 0.216	1.150 ± 0.044	2.313 ± 0.124
TIA(n = 59)	DM(n = 21)	1.171 ± 0.091	4.278 ± 0.144	1.005 ± 0.044	2.387 ± 0.130
	P value	0.148	0.917	0.038	0.703
All(non-AIS,	non-DM(n = 169)	1.459 ± 0.068	4.601 ± 0.085	1.160 ± 0.024	2.427 ± 0.061
n = 225)	DM(n = 56)	1.368 ± 0.073	4.329 ± 0.095	1.070 ± 0.040	2.293 ± 0.075
	P value	0.363	0.035	0.061	0.170
	non-DM(n = 577)	1.467 ± 0.041	4.750 ± 0.042	1.176 ± 0.015	2.513 ± 0.031
All(n = 835)	DM(n = 258)	1.535 ± 0.054	4.677 ± 0.067	1.068 ± 0.020	2.526 ± 0.049
	P value	0.334	0.351	0.000	0.817

### Decreased HDL cholesterol correlated to the AIS with DM

All the patients of AIS, were divided into two groups based on with or without DM. To study the risk factors of AIS with DM, we first performed the single-factor logistic regression, and found that HDL cholesterol was negative correlation to the AIS with DM (OR = 0.373, P = 0.000), while TG, TC, LDL cholesterol have no correlation to it. Furthermore, multinomial logistic regression analysis based on the different HDL cholesterol level demonstrated the OR (95% CI, P value) was 2.113 (1.191-3.749, 0.011) in <1.03 group compared to the reference group (>1.53 group). Also, there were many other factors which influenced the occurrence of AIS with DM, such as Hypertension, BG, UA. Multivariable logistic regression analysis adjusted for Hypertension, BG, UA demonstrated that the same trend was maintained (Table 2).

**Table 2:** **Relative risk of AIS coexistence with DM versus risk factors**

Variable	Beta estimate	Odds ratio	95% CI	P value
Male	-0.205	0.815	0.576-1.153	0.247
Age	0.009	1.009	0.996-1.023	0.175
Hypertension	0.791	2.206	1.467-3.318	0.000
AF	-0.254	0.775	0.463-1.298	0.333
BG	0.594	1.812	1.623-2.023	0.000
UA	-0.003	0.997	0.995-0.999	0.001
TG	0.110	1.117	0.945-1.319	0.194
TC	-0.035	0.965	0.819-1.137	0.672
HDL-C	-0.987	0.373	0.220-0.633	0.000
LDL-C	0.075	1.078	0.860-1.350	0.516
HDL-C(grade)	-	-	-	0.002
>1.53	-	-	-	-
1.03-1.53	0.166	1.180	0.659-2.113	0.577
<1.03	0.748	2.113	1.191-3.749	0.011
HDL-C*	-1.135	0.321	0.166-0.623	0.001
HDL-C(grade)*	-	-	-	0.005
>1.53	-	-	-	-
1.03-1.53	0.323	1.382	0.652-2.928	0.399
<1.03	0.938	2.555	1.212-5.386	0.014

*Multivariable logistic regression, Adjust for Hypertension, BG, UA; HDL-C: HDL cholesterol, LDL-C: LDL cholesterol.

### There has no correlation between HDL cholesterol and AIS

**Figure 2:**
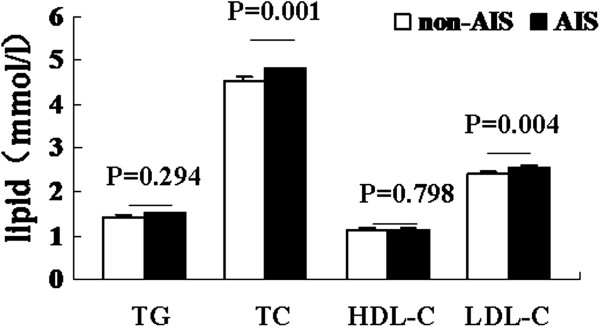
**Comparison of lipid composition between AIS and non-** **AIS patients.** HDL-C: HDL cholesterol, LDL-C: LDL cholesterol.

In the lipid composition, only HDL cholesterol was correlated to the AIS with DM, if this kind of trend was existed in the all AIS patients, which was uncertain. Here, we compared the level of different lipid composition between AIS and non-AIS patients. We found that AIS group had higher level of TC and LDL cholesterol than that in the non-AIS group, but had no significant difference of HDL cholesterol (Figure 2). To further illustrate the risk factors of AIS, we performed logistic regression and found that, there were many factors which would influence the occurrence of AIS such as TC, LDL cholesterol, but not HDL cholesterol (Table 3).

**Table 3:** **Relative risk of AIS versus risk factors**

Variable	Beta estimate	Odds ratio	95% CI	P value
Male	0.457	1.579	1.160-2.151	0.004
Age	0.007	1.007	0.994-1.019	0.290
Hypertension	0.404	1.497	1.086-2.065	0.014
DM	0.402	1.494	1.057-2.112	0.023
AF	7.348	1552.425	0.003-8.1 × 10^8^	0.274
BG	0.436	1.546	1.360-1.759	0.000
UA	0.001	1.001	0.999-1.002	0.241
TG	0.084	1.088	0.916-1.292	0.338
TC	0.257	1.292	1.108-1.507	0.001
HDL-C	0.054	1.055	0.679-1.640	0.812
LDL-C	0.314	1.369	1.106-1.694	0.004

HDL-C: HDL cholesterol, LDL-C: LDL cholesterol.

### Age has the impact on the HDL cholesterol between with and without DM in AIS

Decreased HDL cholesterol was prevalent in the DM group with AIS, which factors would influence this kind of display form? As shown in Table 4, the level of HDL cholesterol was lower in DM group compared to non-DM group both in the populations of aged ≦60 and 61–70 years, but not 71–80 and >80 years. We also found, HDL cholesterol was decreased significantly in the DM group whenever male or female, so did with or without hypertension. The same trend was existed in the AIS without AF, but not in the AIS with AF, which perhaps correlated to the fewer number of patients of the latter (Table 4).

**Table 4:** **Influence factors of HDL cholesterol in the AIS between with or without DM**

Factors	Group				
		≦60(n = 198)	61-70(n = 145)	71-80(n = 183)	>80(n = 84)
Age	non-DM	1.164 ± 0.035	1.166 ± 0.036	1.225 ± 0.034	1.172 ± 0.041
	DM	0.992 ± 0.034	0.993 ± 0.041	1.149 ± 0.040	1.151 ± 0.089
	P value	0.005	0.003	0.154	0.806
		Male(n = 385)		Female(n = 225)	
Sex	non-DM	1.103 ± 0.023		1.327 ± 0.029	
	DM	1.006 ± 0.026		1.159 ± 0.040	
	P value	0.010		0.001	
		Yes(n = 434)		No(n = 176)	
Hypertension	non-DM	1.149 ± 0.020		1.248 ± 0.039	
	DM	1.073 ± 0.026		1.043 ± 0.050	
	P value	0.019		0.010	
		Yes(n = 81)		No(n = 529)	
AF	non-DM	1.322 ± 0.056		1.159 ± 0.019	
	DM	1.156 ± 0.092		1.056 ± 0.023	
	P value	0.123		0.001	

### Low level of HDL cholesterol prevalence in the populations of ≦70 years patients with diabetes and AIS

**Figure 3:**
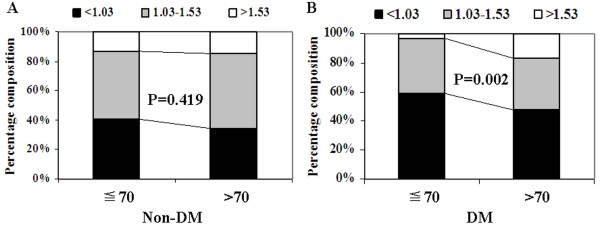
**Distribution of HDL cholesterol levels in the AIS population. (A)** In the populations of Non-DM. **(B)** In the populations of DM.

Both in the AIS patients with and without DM, we analyze the difference of distribution of HDL cholesterol between the populations of ≦70 and >70 years simultaneously, we found that the percent of low level of HDL cholesterol was higher in aged ≦70 years than that in >70 years just in the populations with DM, but not in the non-DM populations (Figure 3).

### Decreased HDL cholesterol correlated to the AIS with DM just in the populations of aged ≦70 years

HDL cholesterol was correlated to the AIS with DM, while age would influence its expression, to illustrate the risk factors of AIS with DM in different age level, we performed the logistic regression respectively in the population of aged ≦70 and >70 years. As shown in Table 5, HDL cholesterol was negative correlation to the AIS with DM just with aged ≦70 years (OR = 0.192, P = 0.000), more high risk was existed in the group of HDL cholesterol <1.03 compared to the >1.53 group (OR = 6.818, P = 0.002). Adjusted for hypertension, BG, UA in multivariable logistic regression analysis, the same trend was still maintained.

**Table 5:** **Relative risk of AIS coexistence with DM versus risk factors in age subgroup**

	≦70 years old(n = 343)		>70 years old(n = 267)	
Variable	OR (95% CI)	P	OR (95% CI)	P value
Male	0.649(0.399-1.057)	0.082	1.104(0.668-1.826)	0.700
Hypertension	2.734(1.542-4.845)	0.001	1.707(0.946-3.078)	0.076
AF	0.509(0.187-1.389)	0.187	0.836(0.446-1,567)	0.576
BG	1.824(1.586-2.097)	0.000	1.847(1.535-2.223)	0.000
UA	0.996(0.993-0.998)	0.001	0.999(0.996-1.001)	0.266
TG	1.093(0.886-1.348)	0.408	1.284(0.950-1.734)	0.104
TC	0.975(0.785-1.212)	0.821	0.971(0.753-1.250)	0.818
HDL-C	0.192(0.085-0.433)	0.000	0.623 (0.298-1.302)	0.208
LDL-C	1.178(0.871-1.592)	0.287	0.984(0.697-1.388)	0.925
HDL-C(grade)	-	0.002	-	0.054
>1.53	-	-	-	-
1.03-1.53	4.049(1.176-13.948)	0.027	0.642(0.307-1.345)	0.240
<1.03	6.818(2.002-23.222)	0.002	1.267(0.609-2.635)	0.527
HDL-C*	0.166(0.058-0.472)	0.001	-	-
HDL-C(grade)*	-	0.016	-	-
>1.53	-	-	-	-
1.03-1.53	4.340(0.827-22.785)	0.083	-	-
<1.03	7.887(1.519-40.959)	0.014	-	-

*Multivariable logistic regression, Adjust for Hypertension, BG, UA; HDL-C: HDL cholesterol, LDL-C: LDL cholesterol.

## Discussion

DM is characterized by hyperglycaemia, but aggressive management of glucose has failed to reduce the incidence of AIS [8–10], which demonstrated that the level of glucose is not the key regulator of the AIS in patients with DM. Also, DM is usually accompany with low plasma HDL cholesterol levels, especially in non-insulin-dependent diabetes [12], while this may not have a direct relationship to degree of glucose control [13, 14]. We wonder whether this kind of low level of HDL cholesterol is correlated to the occurrence of AIS in the DM individuals. In this study, we identified our previous hypotheses, and found that decreased HDL cholesterol was correlated to the occurrence of AIS with DM, which mainly existed in the populations of aged ≦70 years.

According with the previous studies, our finding revealed that low level of HDL cholesterol was prevalent in the DM group, and extended this kind of trend in the populations of AIS and non-AIS. The level of HDL cholesterol was markedly reduced in the populations of diabetes mellitus compared with the non-diabetic controls, which was also confirmed in our study. But in the subgroups, we could see the obvious difference. There was no change of HDL cholesterol between diabetes and non-diabetes in the patients with vertigo or dizziness, which have the cerebrovascular causes only about 4%-6% [15]. But decreased HDL cholesterol has already been seen in the TIA patients, and the manifestation was more prominent in the AIS populations. This seemed to suggested that low level of HDL cholesterol was just existed in the populations of ischemic cerebrovascular or atherosclerotic disease induced by DM, not simple the patients with diabetes.

The association between HDL cholesterol and ischemic stroke was unclear. Generally, an inverse association was supported by a series of prospective cohort studies [5, 6], while no association was reported by the Asia Pacific region Cohort Study [7] and Women’s Health Study [16]. Different results may attribute to define of the target populations. Our finding was consistent with the latter, we did not find any significant association between HDL cholesterol and the risk of total ischemic stroke, but this kind of inverse association was existed between HDL cholesterol and the risk of AIS with DM and low level group of HDL cholesterol had more high risk in the occurrence of AIS with DM than that in the high level group. The exact mechanism behind it was unclear.

Studies on lipid levels and the risk of ischemic stroke were inconsistent. In the past several decades, the main emphases were played on the total cholesterol, HDL cholesterol and LDL cholesterol. As mentioned above, low level of HDL cholesterol was the independent risk factor of AIS with DM, nor total AIS, which just represented our opinion. About the total cholesterol and LDL cholesterol, more evidences demonstrated that increased risk of AIS was associated with increased total cholesterol and LDL cholesterol levels [16–20], while other studies found there had no clear association [21, 22]. In the current study, we found that patients with AIS had high levels of TC and LDL cholesterol which was correlated to the increased risk of ischemic stroke, further identified the previous main reports.

High level of LDL cholesterol was considered as a predictor of ischemic stroke. LDL cholesterol was correlated to the AIS which was confirmed by our present study, strict control of LDL cholesterol was recommended in the prevention of ischemic cerebrovascular disease by many guidelines [23]. But in the development of AIS with DM, we did not find any association between it and LDL cholesterol, while HDL cholesterol played the important role during its whole course. Many guidelines to prevent atherothrombotic diseases also recommend strict control of LDL cholesterol in patients with diabetes [23], should we keep on doing it, or transfer our focus to HDL cholesterol. To make this decision, more studies needed to be done. Perhaps, HDL cholesterol is a new therapeutic target in AIS induced by type 2 diabetes [24].

There are many major determinants for the risk of ischemic stroke, such as age, gender, hypertension, diabetes, lipid and AF. Lipid levels changed obviously in the course of AIS, also diabetes has been proved to have impact on it. What about other factors? Few studies have been done in this field. The association between HDL cholesterol and the risks of ischemic stroke had no difference in varied gender [5], so did with the hypertension or not [25], but the age had the impact on association between HDL cholesterol and the risks of ischemic stroke in diabetic individuals [25]. About the AF, there had no relative reports until now. According with the previously study, we did find the age had the impact on the the level of HDL cholesterol in the AIS populations. That was, lower level of HDL cholesterol was existed in DM group compared to non-DM group both in the populations of AIS with aged ≦60 and 61–70 years, but not 71–80 and >80 years. This might to be the reason of patients with diabetes and ischemic stroke are younger, especially in those patients less than 65 years [26].

Further analyses demonstrated that the populations of aged ≦70 years had higher percent of low level of HDL cholesterol than that in >70 years in the diabetic individuals while not in the non-diabetic individuals. It seemed to suggest that patients with diabetes and aged ≦70 years were more prone to have decreased HDL cholesterol which was correlated to the occurrence of AIS, and we identified it. Decreased HDL cholesterol was the independent risk factor of AIS with DM just in the populations of aged ≦70 years, nor >70 years. In the report of Hayashi T et al. [25], decreased HDL cholesterol was a risk factor for CVD in elderly patients with diabetes subjects including those aged >65 years, especially >75 years. There seemed to be some inconsistence, partly for the difference of age division, others for the difference of analytic populations or else. In our study, more attentions were paid to the decline of HDL cholesterol, based on which can we next analyze the risk relative degree of AIS or AIS with DM. In those aged >70 years, we did not find too much low levels of HDL cholesterol, whenever diabetes or non-diabetes. On the other hand, decreased HDL cholesterol was prevalent in patients with diabetes which played the important role in the AIS, obviously in the populations of aged ≦70 years. Relative results need more studies to confirm.

In summary, our current study provided the evidence that low level of HDL cholesterol was correlated to the occurrence of AIS induced by DM, while other lipid compositions had no impact on it. More important, age had the impact on it. That was, only the populations of aged ≦70 years had this kind of trend. To prevent the occurrence of ischemic stroke in diabetic individuals, more attentions should be paid to the decline of HDL cholesterol, especially those aged ≦70 years.
